# Association of women-specific size threshold and mortality in elective abdominal aortic aneurysm repair

**DOI:** 10.1093/bjs/znad376

**Published:** 2023-11-14

**Authors:** Mareia Talvitie, Magnus Jonsson, Joy Roy, Rebecka Hultgren

**Affiliations:** Department of Molecular Medicine and Surgery, Karolinska Institutet, Stockholm, Sweden; Department of Vascular Surgery, Karolinska University Hospital, Stockholm, Sweden; Department of Molecular Medicine and Surgery, Karolinska Institutet, Stockholm, Sweden; Department of Vascular Surgery, Karolinska University Hospital, Stockholm, Sweden; Department of Molecular Medicine and Surgery, Karolinska Institutet, Stockholm, Sweden; Department of Vascular Surgery, Karolinska University Hospital, Stockholm, Sweden; Department of Molecular Medicine and Surgery, Karolinska Institutet, Stockholm, Sweden; Department of Vascular Surgery, Karolinska University Hospital, Stockholm, Sweden

## Abstract

**Background:**

It is unclear whether women derive mortality benefit from early repair of abdominal aortic aneurysms (AAA). The aim of this study was to compare short- and mid-term mortality for women treated at small *versus* large diameters.

**Method:**

Women receiving elective repair of AAA at small (49–54 mm) and large (≥55 mm) diameters from 2008 to 2022 were extracted from the Swedish National Registry for Vascular Surgery (*n* = 1642 women). The effect of diameter on 90-day, 1- and 3-year mortality was studied in logistic regression and propensity score models. Age, co-morbidities, smoking and repair modality were considered as confounders. Men (*n* = 9047) were analysed in parallel.

**Results:**

Some 1642 women were analysed, of whom 34% underwent repair at small diameters (*versus* 52% of men). Women with small (*versus* large) AAAs were younger (73 *versus* 75 years, *P* < 0.001), and 63% of women in both size groups had endovascular repairs (*P* = 0.120). Mortality was 3.5% (90 days), 7.1% (1 year) and 15.8% (3 years), with no differences between the size strata. There was no consistent association between AAA size and mortality in multivariable models. Sex differences in mortality were almost entirely due to mortality in younger-than-average women *versus* men (3-year mortality: small AAAs 11.1% *versus* 7.3%, *P* < 0.030, or large 14.4% *versus* 10.7%, *P* < 0.038).

**Conclusion:**

Mortality in women is high and unaffected by AAA size at repair. The optimal threshold for women remains undefined. The higher rupture risk in women should not automatically translate into a lower, women-specific threshold.

## Teaser

In this population-based cohort study (utilizing data from the Swedish National Registry for Vascular Surgery 2008–2022), the aim was to investigate whether women treated at small *versus* large abdominal aortic aneurysm diameters (as defined by the threshold for men) gain any mortality benefit from early repair (at 49–54 mm). Among 1642 electively treated women in the nation (of which 34% at small sizes), postoperative mortality was high and unaffected by AAA size at repair. The optimal threshold for women remains undefined, but the known higher rupture risk should not automatically translate into a lowered threshold.

## Background

More evidence is required to strengthen the recommendations for treating women with abdominal aortic aneurysms (AAAs)^1–3^. The study populations in the defining randomized trials^4–8^ (early elective surgery compared to surveillance) conducted some 20 years ago were heavily male-dominated, with women constituting only 0.9–17.2% of the cohorts. Therefore, while the RCT-based recommendation for surgical treatment at 55 mm as indicated in societal guidelines^2,3^ has been well-defined for men, the issue of optimal treatment thresholds for women remains debated and unresolved. This knowledge gap is clearly reflected in the diverging recommendations for women even in most recent guidelines^2,3,9,10^.

The complexity of clinical decision-making for women with AAA is further increased by the known higher risk of rupture under surveillance^11,12^. Women also demonstrate increased risks for complications and mortality, and have impaired long-term relative survival after surgical treatment^13–17^. The causal factors have been nothing but elusive and the identified mortality risks have persisted despite adjustments for various patient-^13,14^ and hospital-level^15^ characteristics. The possibility that women will need an open rather than endovascular repair due to morphological constraints also contributes to the struggle to obtain the safest care at the lowest risk^14,18^.

In order to strengthen the evidence-base and improve the quality of vascular services offered to women with AAAs worldwide, it is necessary to scrutinize the outcomes of women presenting with small and large AAAs at the time of elective treatment (as compared to the current threshold for men, 55 mm). National quality registries provide an excellent platform for such analyses^19^. To date, the topic of mortality rates among women treated at different AAA diameters has only briefly been mentioned in the observational literature^20^. The fact that women could lose morphological endovascular repair (EVAR) eligibility at earlier stages has gained more attention^18^, as well as the concept of larger relative diameters (in relation to body size) among women^21–25^. Still, guidelines remain strongly diameter-based. Comprehensive retrospective analyses are a useful step towards prospective and randomized work within the area.

This study was designed to compare the mortality rates of women electively treated at small (49–54 mm) *versus* large (≥55 mm) AAA diameters by utilizing data from the Swedish National Registry for Vascular Surgery (Swedvasc). Both short- and mid-term mortality rates were assessed. The cohort of men was analysed in parallel.

## Methods

The nationwide cohort of patients undergoing elective surgery for an intact AAA in Sweden during a 14-year period (May 2008–February 2022) was identified in the national quality registry, Swedvasc^26^. The Swedvasc registry has a very high internal and external validity (>95%). Mortality data in Swedvasc are retrieved directly from the Swedish National Population Registry and are nearly 100% accurate^27^. All patients ≥45 years of age with an AAA measuring ≥49 mm were included. Patients undergoing repair for ruptured or symptomatic AAAs were not included. The study was approved by the Swedish Ethical Review Authority (Dnr 2021–01753). The STROBE statement was followed^28^.

### AAA size at repair as exposure

The main exposure was registered AAA size at the time of repair. For women, the AAA was considered small if it measured 49–54 mm. Large AAAs in women therefore measured ≥55 mm. For men, the corresponding measures were defined as 49–59 mm and ≥60 mm. For sensitivity analyses, all patients undergoing surgery for ‘very large’ AAAs were excluded—the sex-specific definitions were >65 mm for women and >70 mm for men.

### Mortality outcomes

The primary outcome was postoperative short- (90-day) and mid-term (1- and 3-year) mortality. The mortality rate is cross-checked monthly in the Swedvasc registry by linking to the Swedish population registry, resulting in registration of the date of certified death within one month post-mortem. Data extraction took place in November 2022; hence, the datafile would include all deaths up to October 2022.

### Variables and cohort

Apart from sex, age and AAA size at repair, data on the following co-morbidities were used (registered diagnosis present at the time of repair): pulmonary disease, heart disease, previous cerebrovascular event, hypertension and diabetes. Smoking was categorized as never or ever (current or previous). For crude analyses, all women and men identified according to the criteria specified above were included. For adjusted analyses, however, a complete-case analysis plan was executed for selected variables: all patients with missing data on any of the first three co-morbidities or smoking were excluded (*Table S1*). The initial datafile (crude data) included 1642 women and 9047 men—after exclusion (adjusted data), 1380 (84%) women and 7515 (83%) men remained.

Treatment modality was registered as open repair or EVAR. Hybrid cases (conversion to open repair, 0.3% for both women and men) were included in the open surgery group.

### Statistical analysis

Baseline demographics were plotted in strata for diameter (and separately for the sexes). Continuous variables were summarized with means with s.d. and ranges or medians; the student’s *t* and Mann–Whitney U tests were used to examine statistical significance. Categorical variables were described as numbers and percentages and differences examined by the Chi^2^ or Fisher’s exact test as appropriate. The study had 80% power to detect a between-group difference of 3–4% in 90-day mortality at the 5%-significance level.

### Logistic regression models

The main analysis strategy had two levels: comparing *women* with small *versus* large AAAs, and comparing *women and men* (while adjusting for diameter stratum and sex). Both comparisons were investigated first by univariate logistic regression models followed by multivariate models yielding final mortality OR for the relative effect of large *versus* small size at repair while adjusting for all other clinically relevant factors (irrespective of their univariate statistical significance). These factors were age (analysed in four categories), co-morbidities (pulmonary disease, heart disease, previous cerebrovascular event), repair modality and smoking status. Logistic regression models were re-run with all time points of interest as outcome (90 days, 1 and 3 years). Sensitivity analyses were conducted where all patients repaired for very large AAAs were excluded. The Kaplan–Meier method was used to depict survival (up to 3 years after repair) and the log-rank test to test differences in survival between the two size categories.

### Propensity score models

As patients undergoing surgery at small instead of large diameters could fundamentally differ with respect to age and co-morbidities, the research objectives (and the two-level analysis strategy of women-only and mixed cohorts) were further investigated with propensity score techniques. A propensity score (for the odds of repair at large compared to small AAA size) was assigned to each patient by means of a logistic regression model that included those variables considered relevant (age [here included as continuous variable], pulmonary disease, heart disease and cerebrovascular event—in addition, patient sex for the mixed cohort). The propensity scores were then utilized for matching and for further logistic regression modelling in the matched sample, while adjusting for the propensity score in the total population, as well as in a pseudopopulation derived from the total population by inverse probability treatment weighting (IPTW) according to commonly used weights. In all of the three resulting models, size at repair was entered as a covariable and repair modality was adjusted for.

For matching, the nearest neighbour technique with a maximal allowed distance of 0.05 was used for 1:1 matching of patients with small and large AAAs. Balanced matching was assessed with histograms and jitter plots of the propensity score distributions, and by assessing the standardized mean differences before and after matching. Mortality rates were compared in the matched sample (McNemar’s test used for statistical assessment; quantile stratification omitted due to small numbers).

Null hypotheses were rejected at *P* < 0.05. Statistical programming was conducted in the R environment (R Core Team [2022], Vienna, Austria). The ‘MatchIt’ package was used for computational matching.

## Results

### Postoperative mortality in cohort of women

During 14 years, a national cohort of 1642 women underwent elective repair for their AAA: just above one-third had small size at the time of repair (34%). Aside from a younger mean age among women repaired for small AAAs (2.1 years, *P* < 0.001), the co-morbidity burdens were similar and the majority (63%) received endovascular repair in both size categories (*P* = 0.120, *Table 1*).

**Table 1:** Demographics, repair modality and mortality rates of women undergoing elective repair at small (49–54 mm) and large (≥55 mm) AAA sizes

	All *n* = 1642	Small *n* = 557 (34%)	Large *n* = 1085 (66%)	*P*
Mean age in years (range, SD)	74.6 (46–94; 6.6)	73.2 (49–90; 6.3)	75.3 (48–94; 6.7)	*<0*.*001*
**Median diameter** in mm (SD)	56 (7.4)	52 (1.5)	60 (7.2)	*<0*.*001*
**Co-morbidity**				
Hypertension	1284 (78.2)	434 (77.9)	850 (78.3)	0.893
Pulmonary disease	521 (31.7)	184 (33.0)	337 (31.1)	0.449
Heart disease	520 (31.7)	164 (29.4)	356 (32.8)	0.183
Cerebrovascular event	194 (11.8)	62 (11.1)	132 (12.2)	0.593
Diabetes	192 (11.7)	68 (12.2)	124 (11.4)	0.701
**Smoking**				
Ever	1162 (70.8)	412 (74.0)	750 (69.1)	
Never	251 (15.3)	80 (14.4)	171 (15.8)	
*Missing*	229 (13.9)	65 (11.7)	164 (15.1)	0.090
**Repair modality**				
Open	610 (37.1)	192 (34.5)	418 (38.5)	
Endovascular	1032 (62.9)	365 (65.5)	667 (61.5)	0.120
**Mortality rate**				
90-day	57 (3.5)	18 (3.2)	39 (3.6)	0.812
1-year	116 (7.1)	26 (4.7)	90 (8.3)	0.090
3-year	260 (15.8)	77 (13.8)	183 (16.9)	0.127

Values are *n* (%) unless otherwise indicated.

**Fig. 1:**
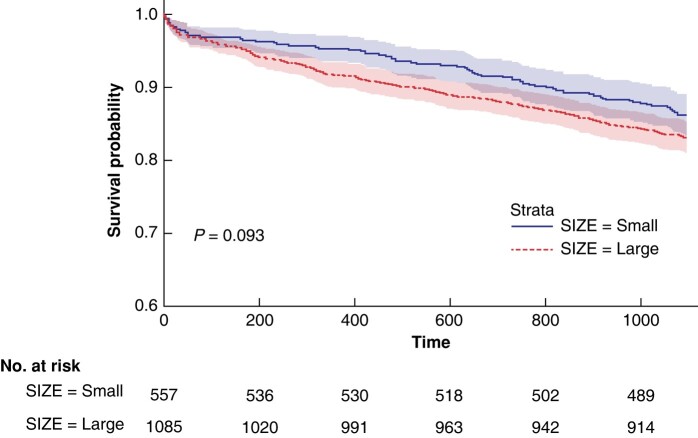
Three-year survival curves (and 95% c.i.s) of women undergoing repair at small *versus* large AAA sizes

Crude 90-day, 1-year and 3-year mortality rates were 3.5%, 7.1% and 15.8%, respectively, and did not differ for women treated at small compared to large diameters (*Table 1*). The similar survival curves of women treated for small and large AAAs is illustrated by the Kaplan–Meier plot in *Fig. 1* (*P* = 0.093).

### Estimated risk in women

In multivariate logistic regression models, AAA size was associated with neither short- nor mid-term mortality (all uni- and multivariate associations collected in *Table S2*). The direction and magnitude of the associations remained near-identical after excluding all women with very large AAAs (*n* = 165). Pulmonary disease greatly increased their risk of 90-day mortality (OR 2.78, 95% c.i. 1.50–5.17, *P* < 0.001, *Table S2*).

After propensity score matching 1:1, the women-only cohort consisted of 940 women. The average mortality rate difference ranged from −0.4% at 90 days (*P* = 0.480) to +2.6% at 3 years (*P* = 0.002) for women undergoing repair at large AAA size (as compared to small AAAs). In the regression models utilizing propensity scores, estimates remained mainly unchanged with no detected effects of size at repair on postoperative mortality. The only exception was 1-year mortality in the IPTW model now predicted by large diameter (OR 1.57, 95% c.i. 1.15–2.15, *P* = 0.005).

### Postoperative mortality in mixed cohort of women and men

Among 9047 men, even numbers of men were treated at small and large diameters (52% and 48%, respectively, *Table S3*). Contrary to women, 90-day, 1-year and 3-year crude mortality were all higher for men repaired for large *versus* small AAAs (1 year: 5.4% *versus* 3.5%, *P* < 0.001, *Table S3*).

In both size categories, women were up to 2 years older than men (*Table S4*). Women with both small and large AAAs had a higher prevalence of pulmonary disease recorded, whereas heart disease was typically recorded as more prevalent among men. Treatment modality distributions were similar between women and men, both overall and in size strata.

**Fig. 2:**
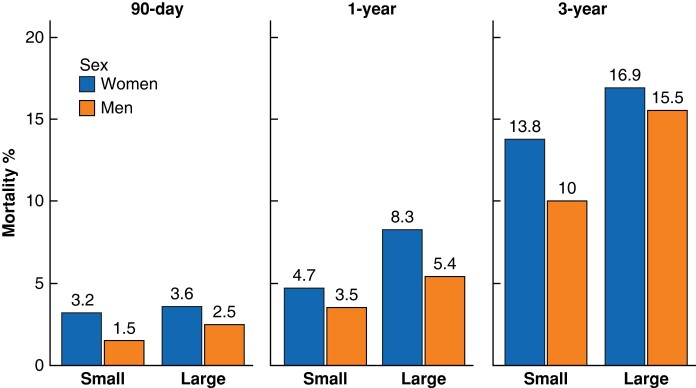
Bar plot of crude short- and mid-term mortality rates of women and men repaired for AAA at small and large sizes

Overall, all crude mortality rates were higher for women than men (*Fig. 2*). Apart from 1-year mortality, these differences resulted from the cohort of small AAAs (*Table S4*). Among patients repaired for small AAAs, sex differences in mortality only remained for EVAR-treated patients (90-day mortality women *versus* men after EVAR for small AAA: 3.6% *versus* 1.1%, *P* < 0.002 [*n* = 303 women and 2346 men], all comparisons for open repair not significant, data not shown). After further stratification for age (cutoff point 73 years = mean age of women treated for small AAAs), sex differences in crude mortality were almost exclusively present in the younger age category (*Table 2*). When examining these younger patients, sex differences were exclusively seen after EVAR (no time comparisons significant in open group, data not shown). The mortality was especially high for younger-than-average women, treated with EVAR, at small sizes (90-day mortality 5.5% for these 128 women, *versus* 1.0% among 1143 men, *P* < 0.002; 3-year mortality 15.6% *versus* 8.5%, *P* < 0.013, data not shown).

**Table 2:** Short- and mid-term mortality rates of women and men, in two age groups and stratified by AAA size at repair

Age (years)	Small			Large		
**≤73**	**Women = 280**	**Men = 2752**	***P***	**Women = 403**	**Men = 2079**	***P***
90-day	9 (3.2)	31 (1.1)	*0*.*009*	11 (2.7)	42 (2.0)	0.476
1-year	13 (4.6)	77 (2.8)	0.122	28 (6.9)	86 (4.1)	*0*.*019*
3-year	31 (11.1)	200 (7.3)	*0*.*030*	58 (14.4)	222 (10.7)	*0*.*038*
**>73**	**Women = 277**	**Men = 1920**	***P***	**Women = 682**	**Men = 2296**	***P***
90-day	9 (3.2)	37 (1.9)	0.225	28 (4.1)	66 (2.9)	0.136
1-year	13 (4.7)	86 (4.5)	0.996	62 (9.1)	150 (6.5)	*0*.*028*
3-year	46 (16.6)	266 (13.9)	0.257	125 (18.3)	456 (19.9)	0.407

The cutoff point of 73 years was chosen to represent the mean age of women treated for small AAAs. Significant differences have been highlighted. Values are given as *n* (%).

### Estimated risk in mixed cohort

When the mixed-sex cohort (1380 women and 7515 men in adjusted analyses) was investigated in logistic regression models, large size was associated with increased mortality at all time points (36% to 46% increased adjusted odds; all uni- and multivariate associations are found in *Table S5*). Excluding all women (*n* = 165) and men (*n* = 795) with very large AAAs eliminated the effect of size on 90-day mortality.

After propensity score matching 1:1, the mixed cohort consisted of 7462 patients. The average mortality rate difference was 0.6–2.6% higher for patients undergoing repair at large AAA size (90 days to 3 years, *P* < 0.001 for all comparisons, data not shown). In the regression models utilizing propensity scores, 90-day mortality was not predicted by diameter in the matched sample, similar to the sensitivity analysis that excluded very large AAAs. In all other models using propensity scores, the estimates remained unchanged with size at repair affecting mortality risks.

*Table 3* is a summary table of all the mentioned associations between AAA size at repair and mortality. When women and men were investigated together in a mixed cohort, large size was a consistent risk factor for short- and mid-term mortality (13/15 models employed with detected association, *Table 3*). However, when only women were analysed, large size was not a risk factor for mortality except in one single model (14/15 models employed without detected association, *Table 3*).

**Table 3:** Summary table of the association between AAA size at repair and mortality

	90-day mortality		1-year mortality		3-year mortality	
	Multivariate OR (95% c.i.)	*P*	Multivariate OR (95% c.i.)	*P*	Multivariate OR (95% c.i.)	*P*
Small size	Ref		Ref		Ref	
*Logistic regression*						
**Women**						
Large	0.89 (0.48–1.64)	0.705—ns	1.51 (0.93–2.47)	0.093—ns	1.16 (0.84–1.60)	0.367—ns
Large but not very	0.91 (0.48–1.72)	0.768—ns	1.43 (0.87–2.37)	0.162—ns	1.08 (0.78–1.51)	0.633—ns
**Mixed cohort**						
Large	1.46 (1.07–1.98)	0.016*	1.52 (1.23–1.87)	<0.001*	1.36 (1.20–1.56)	<0.001*
Large but not very	1.34 (0.97–1.87)	0.080—ns	1.47 (1.18–1.84)	<0.001*	1.31 (1.14–1.51)	<0.001*
*Propensity score*						
**Women**						
Large, adjusting for PS	0.91 (0.49–1.68)	0.763—ns	1.57 (0.96–2.55)	0.070—ns	1.16 (0.84–1.59)	0.369—ns
Large, PS-matched	0.85 (0.42–1.72)	0.648—ns	1.59 (0.92–2.74)	0.096—ns	1.30 (0.91–1.87)	0.154—ns
Large, IPTW	0.93 (0.62–1.39)	0.713—ns	1.57 (1.15–2.15)	0.005*	1.16 (0.94–1.43)	0.168—ns
**Mixed cohort**						
Large, adjusting for PS	1.42 (1.05–1.94)	0.025*	1.53 (1.24–1.88)	<0.001*	1.36 (1.20–1.55)	<0.001*
Large, PS-matched	1.37 (0.98–1.91)	0.062—ns	1.48 (1.19–1.86)	<0.001*	1.30 (1.13–1.49)	<0.001*
Large, IPTW	1.41 (1.14–1.74)	<0.001*	1.53 (1.32–1.77)	<0.001*	1.36 (1.24–1.49)	<0.001*

Logistic regression and propensity score models. For clarity, significant associations have been marked with an asterix (*), and non-significant with ‘ns’. PS, propensity score; IPTW, inverse probability treatment weighting.

## Discussion

This population-based observational appraisal failed to show any mortality benefits for women treated at a lower size threshold. The high mortality in women persisted regardless of AAA size, and seemed to exceed what is expected on the basis of contemporary case mix series^29,30^—only 85% remained alive at 3 years after repair. Even more concerning was that the high mortality was not limited to elderly women treated at large diameters; rather, younger women (below mean age) suffered high mortality rates and contributed the most to differences observed between the sexes.

The threshold for surgical repair in men with AAA has been determined in pivotal RCTs. The high rupture risk in women, combined with women’s smaller stature, has resulted in a soft modification and lowering of this threshold to 50 mm. There is little scientific evidence to support this modification. However, such new thresholds would be supported by a possible survival benefit in women treated early at smaller sizes, or alternatively, a considerable expected reduction in rupture rates. The findings of the current study imply that early repair carries no such mortality reduction for women—not even for younger women—that would justify a general treatment recommendation of AAAs at a lower threshold.

These data add to a multitude of reports that have repeatedly shown worse elective outcomes for women in clinical settings spread across time and locations^13–16,24,31,32^. The sex-specific mean ages and prevalence estimates of concomitant diseases show characteristics typically seen in AAA cohorts^14–16,33,34^.

Vascular physicians face difficult risk–benefit assessments when treating women with AAA. The decision to treat any woman (or man) with an AAA is dependent on two risks: the risks of rupture without repair, and the mortality risk associated with repair. In order to improve treatment decisions and reduce AAA-related mortality among women, a more solid scientific understanding of these two risks has to be developed. The poorly defined risk among women underlies the disagreement in prevailing recommendations and suggested repair thresholds for women, as stated by the European Society for Vascular Surgery^2^ (≥50 mm), Society for Vascular Surgery^3^ (50–54 mm) and NICE^10^ (≥55 mm) guidelines. The extrapolation of data from male-dominated cohorts to women is a simplification of available evidence, a safety-driven rationale that could now be causing more harm than good.

The sex disparity in rupture risk is well known: 13–20% of women rupture at or below 55 mm compared to 5–12% men^24,35^, and multiple studies (with mixed modalities of ultrasound and CT) have found roughly 1 cm smaller mean diameters at rupture for women^12,17,20,24,36–38^. Empirically, ruptures at very large diameters in women are rarely seen^35^. One of the few if not the only available rupture rate estimate for women with small AAAs (identical definition of 50–54 mm) comes from the study by Lancaster *et* al.^39^ (*n* = 567 women): 3.4% at 3 years (95% c.i. 2.1–5.1). This could mean that only at 3 years is the rupture risk comparable to the 90-day mortality of women undergoing planned repair: 3.5% for all women and 3.2% for younger women (≤73 years) in the current study.

When men were included, large diameter did influence both short- and long-term mortality in this report. The composition of the male cohort is most likely very different to that of women due to treatment selection bias: women are much less likely to undergo elective repair^11,14,40–43^ and, thus, to be included in the registry at all. The proportion of men undergoing postponed surgery at an individualized, higher threshold due to severe co-morbidities or complicating anatomical factors is almost certainly higher^29^. This means that any association between diameter and mortality for men could be produced by residual confounding. In a sensitivity analysis, all men with very large diameters were excluded—90-day mortality was then no longer associated with diameter at repair.

The observational literature on the effect of diameter on mortality after elective AAA repair is relatively sparse or almost non-existent (women). Diameter group definitions have varied, and the focus has been on studying the effect in mixed cohorts, with female sex entered as a covariable in statistical models^29,44–46^. For open repairs, Mehta *et al*.^44^ investigated the association in a mixed-sex cohort and found that every 10-mm increase in diameter entailed a higher risk for 30-day (18%) and 1-year mortality (18%). In a large mixed cohort from the VQI database on endovascular repairs^45^, it was concluded that the mortality increased in the group of AAAs >55 mm. After matching for propensity scores, 30-day mortality was very low and similar in all size groups. In the EUROSTAR registry^46^, patients in the largest size group (≥65 mm; only 5–7% women in all size groups) had longer operative times and experienced more perioperative additional procedures. The independent risks were elevated for aneurysm-related mortality (HR 2.6–6.0 for early and late), post-EVAR ruptures (HR 7.7) and type I endoleaks. The only study to use sex-specific definitions of small AAAs^29^ reported longer operative times, more frequent type I endoleaks and a higher hazard for mortality up to 5 years (50–75%). Again, risks only applied for patients with the largest AAAs, corresponding to the ‘very large’ aneurysms in women in the current study. Khashram *et al*.^47^ summarized in a systematic review and meta-analysis on 19 700 patients (from 16 studies) that preoperative diameter, as a continuous variable, increased the risk of long-term mortality by 14% (1.10–1.18), even more so for EVAR than OR (20% *versus* 8%). All these data are well aligned with the present findings, but less helpful for analysing the effect in women, especially as 9/16 of the studies in the meta-analysis^47^ had cohorts with ≥88% men.

There is no replacement for RCTs that have the unique potential to inform clinical guidelines, decisions and best practices. Still, quality registries and national data sets with high internal and external validity are an integral part of the evidence base. It is somewhat surprising that the conclusions from the AAA RCTs, which suffered from the shortcoming of underrepresenting women, have not been challenged or revisited in observational research over the years.

There is a special notation in the Society for Vascular Surgery guidelines^3^ regarding the recommendation to treat women, where the consideration of early repair is especially encouraged in the case of ‘young and healthy patients’. There is also a widely accepted consensus in clinical vascular care that women’s older age is a main factor behind the observed worse outcomes. One of the most important findings of this paper is that for women, young age and small aneurysms do not provide protection against high mortality rates. In fact, sex differences in survival after repair were almost entirely explained by survival discrepancies among younger patients (≤73 years). These facts have fundamental implications considering the very high proportions of women treated at small diameters (<55 mm) in the VSGNE (43%)^21^ and VASCUNET databases (9–48%, with 20% of the women with highest perioperative risk scores treated at small sizes)^48^.

These observational results strongly call for multinational randomized efforts, such as the upcoming WARRIOR Trial (early EVAR *versus* routine surveillance for women), to support guideline-indicated optimal treatment thresholds in women with AAA. Sex differences in mortality after elective AAA surgery, as witnessed for decades, seem to stem from outcome discrepancies among younger patients, providing some novel insight and research leads for future work.

The external and internal validity of the Swedish national quality registry for vascular procedures has been high (98.8% and 96.2%, respectively)^19,26,27^. Previous internal validation has included the diameter variable^26,27^, and a core lab was not used for further validation within the scope of this study.

This is an observational study, and there could be unrecognized factors that influence patient selection for early repair. A causal factor that would go undetected in the current analysis is that women could to a larger extent be suffering from multilevel disease. There is quite strong evidence that AAA disease manifests differently in women, with a higher prevalence of concomitant thoracic aortic aneurysms in women^49,50^ and a greater representation of women in series investigating complex endovascular solutions^50,51^. There is also a possibility of unrecognized and undertreated cardiac and vascular conditions in the cohort of women. Cause of death data would be valuable but were not available for analysis in the current data set.

Pulmonary disease was a strong risk factor for mortality in almost all regression models, although more specific information including data on disease classification^52^ was not available. Statin and antiplatelet therapy have led to reductions in overall and postoperative AAA mortality^53,54^, yet were not adjusted for here due to inaccessible data. Cases with missing values on smoking or chosen co-morbidities were included in crude data but excluded in regression models and, as such, values in the latter analyses were presumed to be missing at random. While a comprehensive national population was investigated in this paper and as many as 560 women with small AAAs were investigated, this group still represents a minority with results dependent on changes in study power.

## Supplementary Material

znad376_Supplementary_Data

## Data Availability

Data will be made available upon request.
