# Supplementary material for: Association of women-specific size threshold and mortality in elective abdominal aortic aneurysm repair
Source: Br J Surg. 2023 Nov 14;111(1):znad376. doi: 10.1093/bjs/znad376 (PMC10776526; doi:10.1093/bjs/znad376)
Supplement: znad376_Supplementary_Data [file znad376_supplementary_data.docx]

Association of Women-Specific Size Threshold and Mortality in Elective Abdominal Aortic Aneurysm Repair

Talvitie Mareia ^1,2^

Jonsson Magnus ^1,2^

Roy Joy ^1,2^

Hultgren Rebecka ^1,2^

^1^Department of Molecular Medicine and Surgery, Karolinska Institutet, Stockholm, Sweden

^2^Department of Vascular Surgery, Karolinska University Hospital, Stockholm, Sweden

**Corresponding author.** Rebecka Hultgren. ME Kärlkirurgi, Norrbacka S3:01, Karolinska Universitetssjukhuset 171 64 Solna, Sweden **ORCID ID** 0000-0002-8869-0493

**Supplementary Materials - Index**

| **Supplementary Tables** |  |
| --- | --- |
| Tables S1-S5 | *page 2-6* |

**Supplementary Figures and Tables**

**Table S1.** Specification of numbers with missing variables, subsequently excluded from the study (complete-case analysis).

| **Missing variable** | **All = 10 689** | **Men = 9047** | **Women = 1642** |
| --- | --- | --- | --- |
| Smoking | 1539 (14.4) | 1310 (14.5) | 229 (13.9) |
| Pulmonary disease | 399 (3.7) | 336 (3.7) | 63 (3.8) |
| Heart disease | 284 (2.7) | 232 (2.6) | 52 (3.2) |
| Cerebrovascular event | 396 (3.7) | 333 (3.7) | 63 (3.8) |

Values are given as n (%).

**
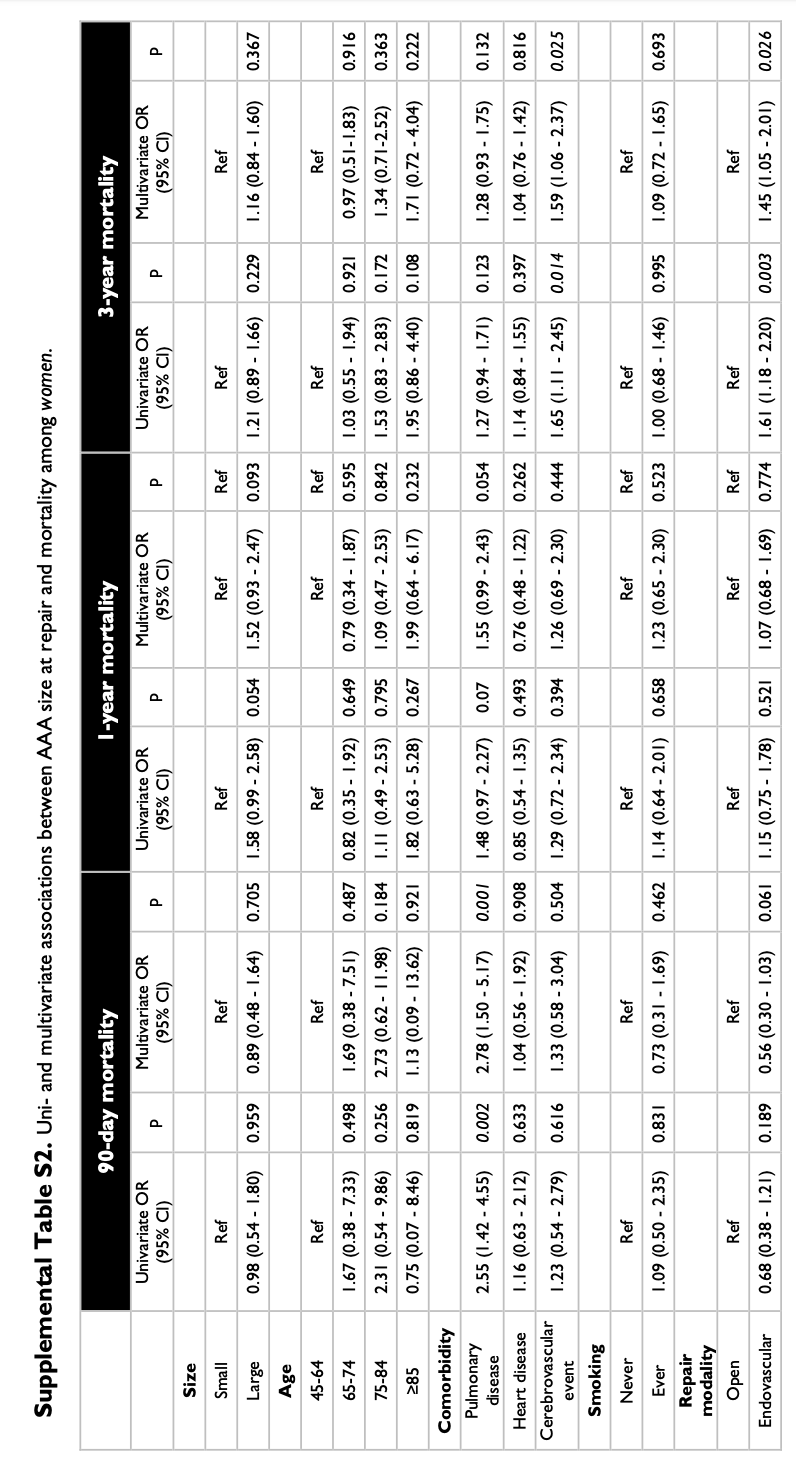
**

**
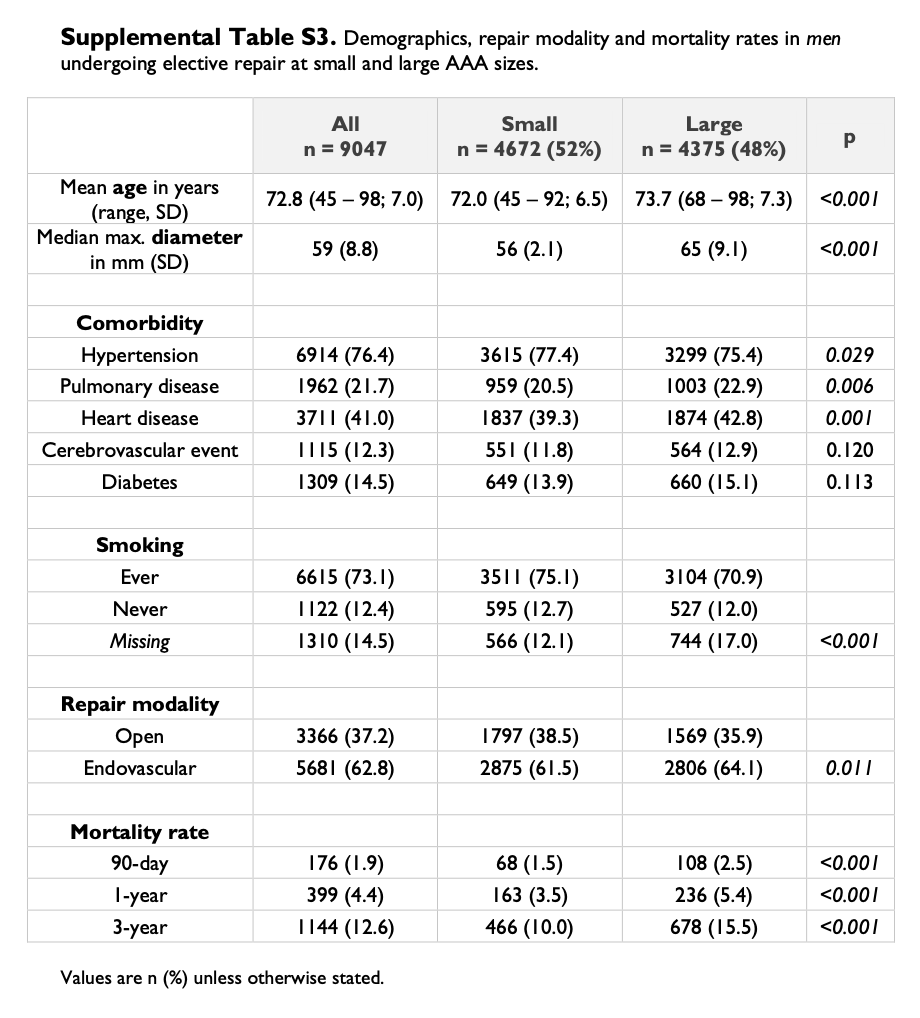
**

**
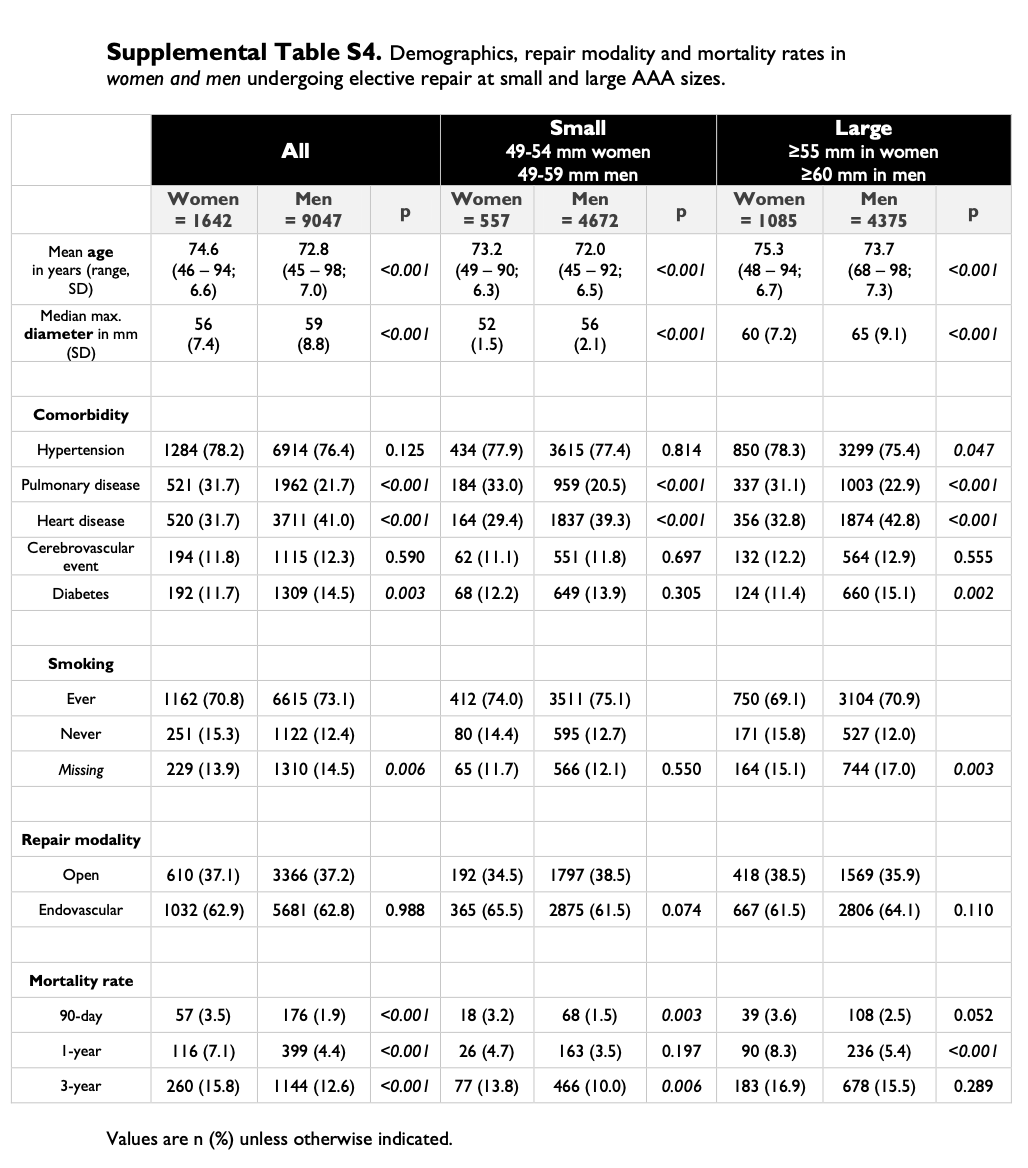
**

**
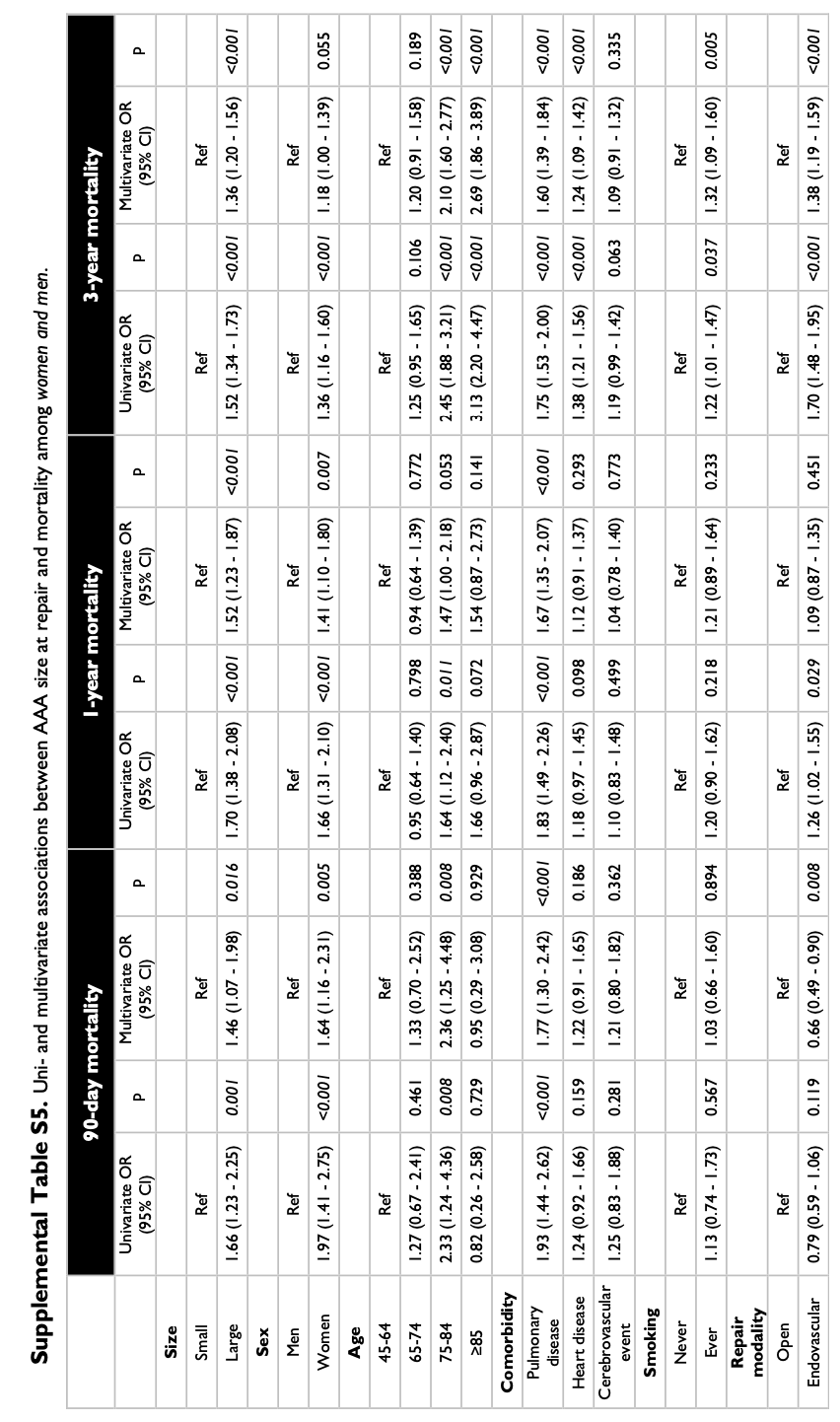
**
